# Reduced latency in manual interception with anticipatory smooth eye movements

**DOI:** 10.1016/j.isci.2025.111849

**Published:** 2025-01-20

**Authors:** Takeshi Miyamoto, Kosuke Numasawa, Riku Hirano, Yusei Yoshimura, Seiji Ono

**Affiliations:** 1 Graduate School of Informatics, Nagoya University, Aichi, Japan; 2 Faculty of Health and Sport Sciences, University of Tsukuba, Ibaraki, Japan; 3 Graduate School of Comprehensive Human Sciences, University of Tsukuba, Ibaraki, Japan

**Keywords:** Neuroscience, Sensory neuroscience

## Abstract

Delays in visuomotor processing can cause the image of a moving object to fall outside the limited range of central vision, resulting in a blurred perception. To overcome these delays, anticipatory smooth eye movements (ASEMs) occur in the expected direction of future object motion before the object begins to move. This study demonstrated the functional benefits of ASEMs in rapid visually guided behaviors, highlighting their role beyond merely compensating for visuomotor delays. By experimentally facilitating ASEMs independent of participants’ predictions regarding future object motion, we showed that ASEMs themselves expedite the initiation of interception movements. Our results revealed a quantitative relationship between ASEM velocity and interception latency on a single-trial level, which was more pronounced in participants employing predictive control compared to those relying on reactive control. These findings suggest that ASEMs enhance the feedforward control of interception movements by providing extraretinal signals rather than retinal signals.

## Highlights

• Anticipatory smooth eye movements (ASEMs) were manipulated during interception
• Facilitating ASEMs reduced interception latency without altering hand trajectory
• This effect was pronounced in ones employing predictive control for interception

## Graphical abstract

**Figure:**
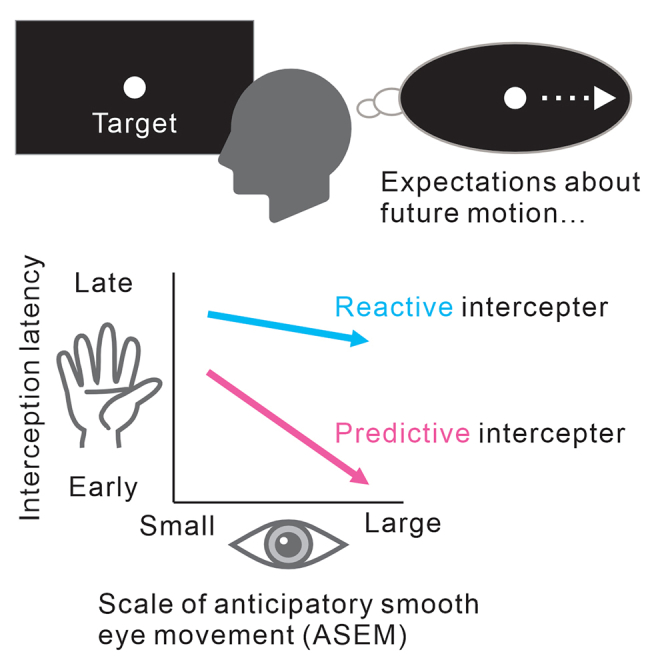

## Introduction

Overcoming sensorimotor delays is a universal challenge for biological motor systems seeking to achieve optimal behaviors. In the visuomotor process, delays of approximately 100 ms can critically impact the perception of visual motion, primarily due to the limited range of central vision.^1^ To address this functional problem, the oculomotor system generates ocular tracking, known as smooth pursuit eye movements, before an object of interest begins to move. This phenomenon is referred to as anticipatory smooth eye movements (ASEMs).^2^^,^^3^ Since its conceptualization,^4^^,^^5^ growing evidence suggests that ASEMs are driven by inferences about object motion based on the weighting of multiple sources of information, rather than solely by motor priming to past stimuli.^2^^,^^6^^,^^7^^,^^8^ This adaptability in ASEM control suggests the importance of early visual information and ocular tracking in visually guided behaviors when interacting with a moving object. Indeed, our own research has demonstrated that experimental facilitation or inhibition of ASEMs can bias the perceived velocity of object motion, even when the steady-state eye velocity after catch-up remains identical across conditions.^9^

Despite the widely recognized importance of eye-hand coordination in various skilled actions,^10^ little is known about whether ASEMs benefit subsequent or ongoing limb motor control when interacting with a moving object. While the relationship between hand movements and smooth pursuit is not as well understood as that with saccades, smooth pursuit has been observed during manual interception of a moving object in both humans and monkeys.^11^^,^^12^ The functional roles of smooth pursuit in this context may include continuously sampling visual information and extrapolating object trajectories during periods of temporal visual occlusion.^13^^,^^14^^,^^15^ If eye movements that align functionally with the desired action influence motor performance, ASEMs are expected to impact rapid limb movements, such as those involved in intercepting a moving object, which has been highlighted in the context of saccades. Studies on saccades have shown that hand movements are modulated by both retinal^16^^,^^17^ and extraretinal^18^^,^^19^ signals from eye movements. Given that the consistency of eye-hand coordination is maintained whether hand and eye movements are discrete or continuous,^20^ findings related to saccades would be applicable to ASEMs. For example, a change in visual speed perception due to ASEMs, induced by a reduction in retinal slip velocity at the onset of object motion,^9^ could modulate the interception trajectory. Furthermore, given the correlation between saccadic and hand reaction times,^11^^,^^21^^,^^22^^,^^23^ early initiation of smooth pursuit could expedite the onset of interception movements.

Knowledge about the impacts of ASEMs on visual perception and limb motor control is less developed compared to other eye movements. This is partly because experimental paradigms used to induce ASEMs often simultaneously affect eye movements, visual perception, and motor planning, making it difficult to isolate the effect of ASEMs alone. For instance, expectations about the future state of a target are a common factor regulating ASEMs,^24^^,^^25^ visual motion perception,^26^^,^^27^ and control of reaching,^28^^,^^29^ but the changes in each of these systems could, at least in part, reflect their interactions. In the present study, we employed a methodology similar to that of our previous research^9^ to test the direct impacts of ASEMs themselves on rapid interception movements, independent of cognitive factors including participants’ expectations regarding future object motion. Our results showed that experimentally facilitating ASEMs expedited the initiation of interception movements. Further analysis revealed a quantitative relationship between ASEM velocity and interception latency on a single-trial level, which varied based on participants’ interception strategies. Notably, interception latencies in participants employing predictive control were more sensitive to ASEM velocity, with the variance in interception latency better explained by ASEM velocity, compared to those relying on reactive control. Our findings provide evidence that ASEMs not only help compensate for the visual information loss at the onset of object motion but also expedite the initiation of rapid visually guided behaviors.

## Results

**Figure 1:**
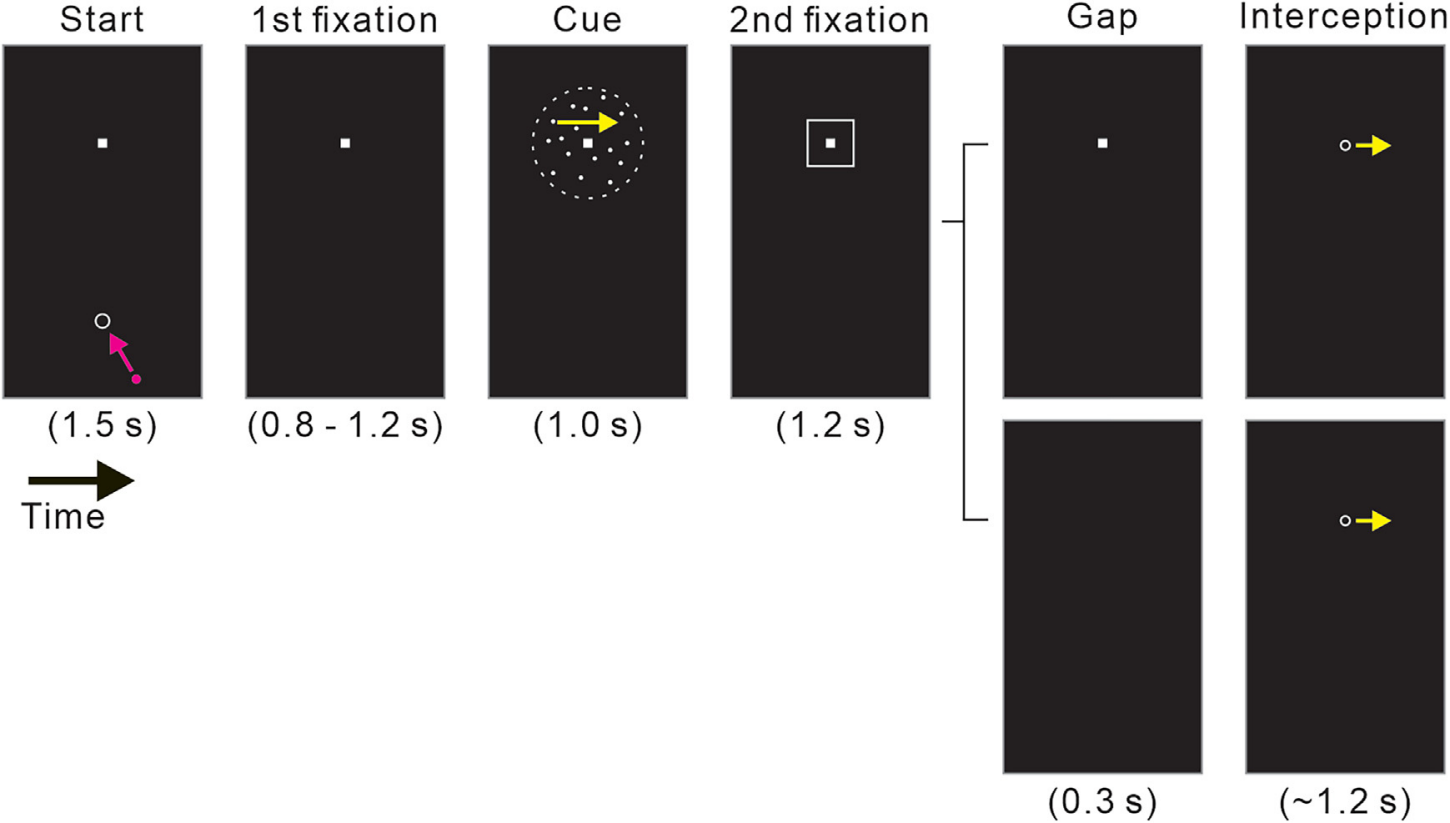
Trial sequence and conditions Each trial started with placing a magenta cursor within a starting circle while gazing at a fixation spot (“Start” period). After a random delay of 0.8–1.2 s (“1st fixation” period), a random-dot motion (RDM) stimulus appeared within a 15 deg diameter aperture centered on the fixation spot for 1.0 s (“Cue” period). Subsequently, the fixation spot and a square (5.0 × 5.0 deg) surrounding it were presented (“2nd fixation” period). In the control condition, only the square disappeared after 1.2 s while the fixation spot remained for an additional 0.3 s (upper panel in “Gap” period). In the gap condition, both the fixation spot and the square disappeared after 1.2 s, followed by a 0.3 s gap period (lower panel in “Gap” period). At 1.5 s after the onset of the second fixation period, a white ring target appeared at the location of the fixation spot and then moved at a constant speed of 8.0 deg/s in the same direction as the coherent motion of the RDM stimulus presented in the cue period (“Interception” period). Participants were instructed to maneuver an invisible cursor on a monitor, controlled by a stylus, to intercept the target as quickly as possible. Arrows depict examples of cursor manipulation (magenta arrow in the “Start” period), the directions of coherent motion of dots (yellow arrow in the “Cue” period), and the target (yellow arrow in the “Interception” period). These arrows are meant to help readers understand the task events and were not presented during the experiment.

Figure 1 illustrates the experimental procedure (see the STAR Methods section for details). Participants were instructed to maneuver an invisible cursor on a monitor, controlled by a stylus, to intercept a visual target moving horizontally at a constant velocity of 8.0 degrees/second (deg/s) (represented as the white ring in “Interception” period in Figure 1). The direction of the target motion was aligned with the direction of coherent motion in a random-dot motion (RDM) stimulus presented prior to the target’s appearance (“Cue” period in Figure 1). Thus, the direction of coherent motion in the RDM stimulus determined the target’s motion direction (either right or left), and its coherence level dictated the level of certainty in motion prediction. We designed two experimental conditions to either facilitate or suppress ASEM responses. This was achieved by varying the presentation of a fixation spot before the onset of target motion: the fixation was released in the gap condition but remained in the control condition (“Gap” period in Figure 1). The gap period, which occurs immediately before target motion onset, is known to facilitate ASEM responses.^30^^,^^31^^,^^32^ This manipulation potentially provided an additional cue regarding the timing of target motion onset only in the gap condition (i.e., the disappearance of the fixation spot indicated that the target motion would begin 0.3 s later). To ensure that the knowledge of onset timing of target motion was equivalent across both conditions, a square surrounding the fixation spot was presented in the same manner as in the gap condition. That is, the square was displayed together with the fixation spot and then was removed 0.3 s before target motion onset in all trials (“2nd fixation” period in Figure 1). These procedures ensured that cognitive factors, such as prediction of motion direction and target motion onset timing, were consistent across both conditions, allowing for a direct examination of the impact of ASEMs on interception movements.

### Eye movement behaviors

**Figure 2:**
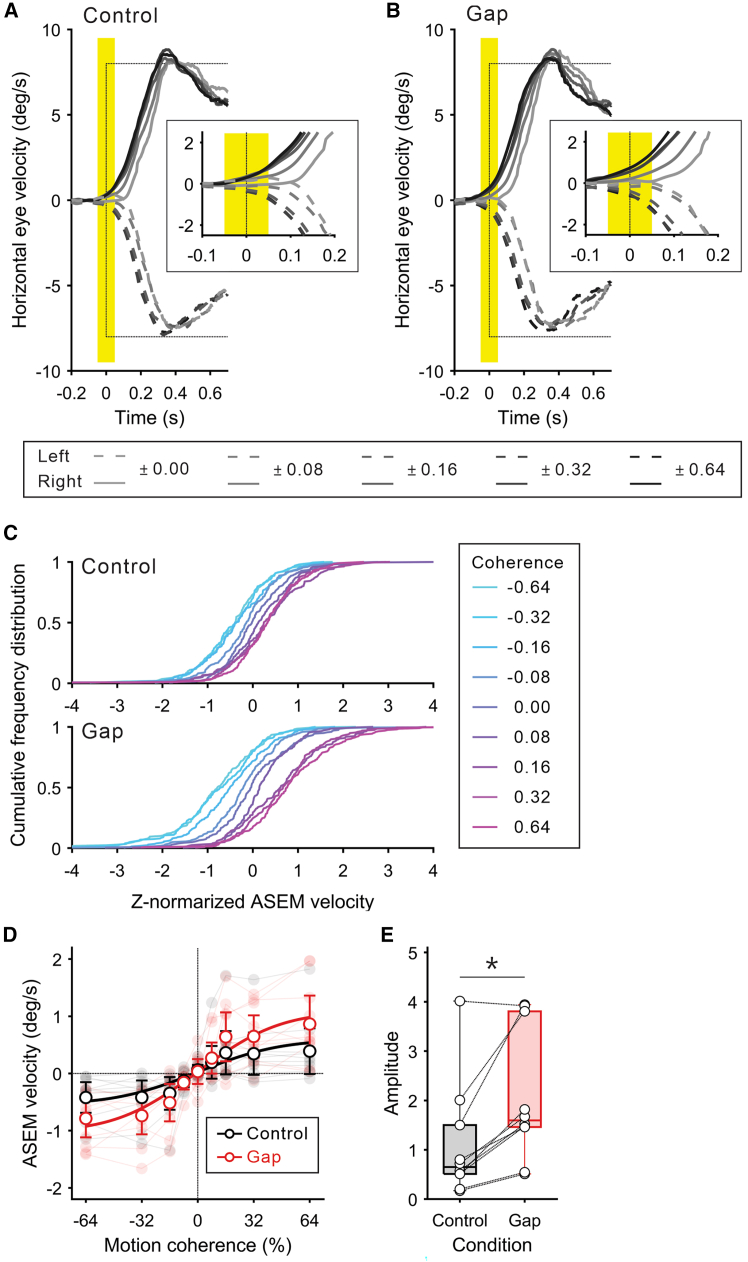
Eye movement behaviors (A) Mean traces of horizontal eye velocity across all participants (*n* = 10) for the control condition. Time 0 denotes the onset of target motion. Solid and dashed lines indicate rightward and leftward eye movements, respectively. Dotted lines indicate target velocity in both directions. Shading indicates the period used to compute ASEM velocity. The inset shows the enlarged view around the onset of target motion. (B) Mean traces of horizontal eye velocity across all participants for the gap condition, plotted in the same format as Figure 2A. (C) Cumulative frequency distribution of ASEM velocity. The upper and lower panels indicate the distribution for the control and gap conditions, respectively. Data were z-normalized across all trials for each participant and then pooled. (D) ASEM velocity as a function of motion coherence. Data are represented as mean and 95% confidence interval. Pale lines and dots in the background show individuals’ data. Solid curves indicate cumulative Gaussian functions fitted to the mean data for each condition. (E) Height of the cumulative Gaussian function shown in Figure 2C. Circles connected by dotted lines denote individuals’ data. An asterisk indicates a significant difference between conditions (paired *t* test: *t*_9_ = −4.10, *p* = 2.70 × 10^−3^, Cohen’s *d* = 0.74).

As expected, eye velocity varied according to the level of prediction certainty (Figures 2A and 2B). Specifically, ASEM velocity, defined as the mean eye velocity within a time window spanning 50 ms before and after the target motion onset, was modulated as a function of prediction certainty.^3^^,^^27^^,^^33^ This was observed even in the control condition in which ASEM responses were suppressed. This is because the participants were aware of the target motion onset timing due to the consistent length of the fixation period before target motion onset throughout the experiment, which is a key factor in inducing ASEMs.^24^^,^^25^ We examined the distribution of ASEM velocity for each combination of ASEM condition and motion coherence level to investigate whether the decision outcome (i.e., left or right) or the confidence level in the perceptual decision regarding the RDM stimulus influenced ASEM velocity. If ASEM velocity was determined by the outcome of their decision, we would expect a bimodal distribution corresponding to the two alternatives. Conversely, if ASEM velocity was determined by the decision confidence, the distribution would remain unimodal but shift according to the level of prediction certainty. The result revealed a unimodal distribution in all combinations (Figure 2C), suggesting that ASEM velocity reflects participants’ confidence in their perceptual decisions rather than the outcome of their decisions. This modulation of ASEM velocity as a function of prediction certainty aligns with the principles of a Bayesian decision model, where observers form a belief about future target motion based on their prediction certainty and transform this belief into action.^34^ Consequently, ASEM velocity dependent on motion coherence was well described by a sigmoid curve (cumulative Gaussian function), with mean *r*^2^ values of 0.64 (SD: 0.23) for the control condition and 0.83 (SD: 0.11) for the gap condition (Figure 2D), consistent with previous studies on perceptual decision-making in response to RDM stimuli.^35^^,^^36^^,^^37^ The gap condition induced larger ASEM velocity (ASEM velocity in the target motion direction: 0.28 ± 0.34 deg/s for the control condition, 0.52 ± 0.35 deg/s for the gap condition, mean ± SD; Figure S1), as reflected in the height of the cumulative Gaussian function (*t*_9_ = −4.10, *p* = 2.70 × 10^−3^, Cohen’s *d* = 0.80; Figure 2E). These results confirmed that our task conditions altered ASEM responses even at identical levels of prediction certainty.

### Facilitating ASEMs reduces interception latency while maintaining hand trajectory

**Figure 3:**
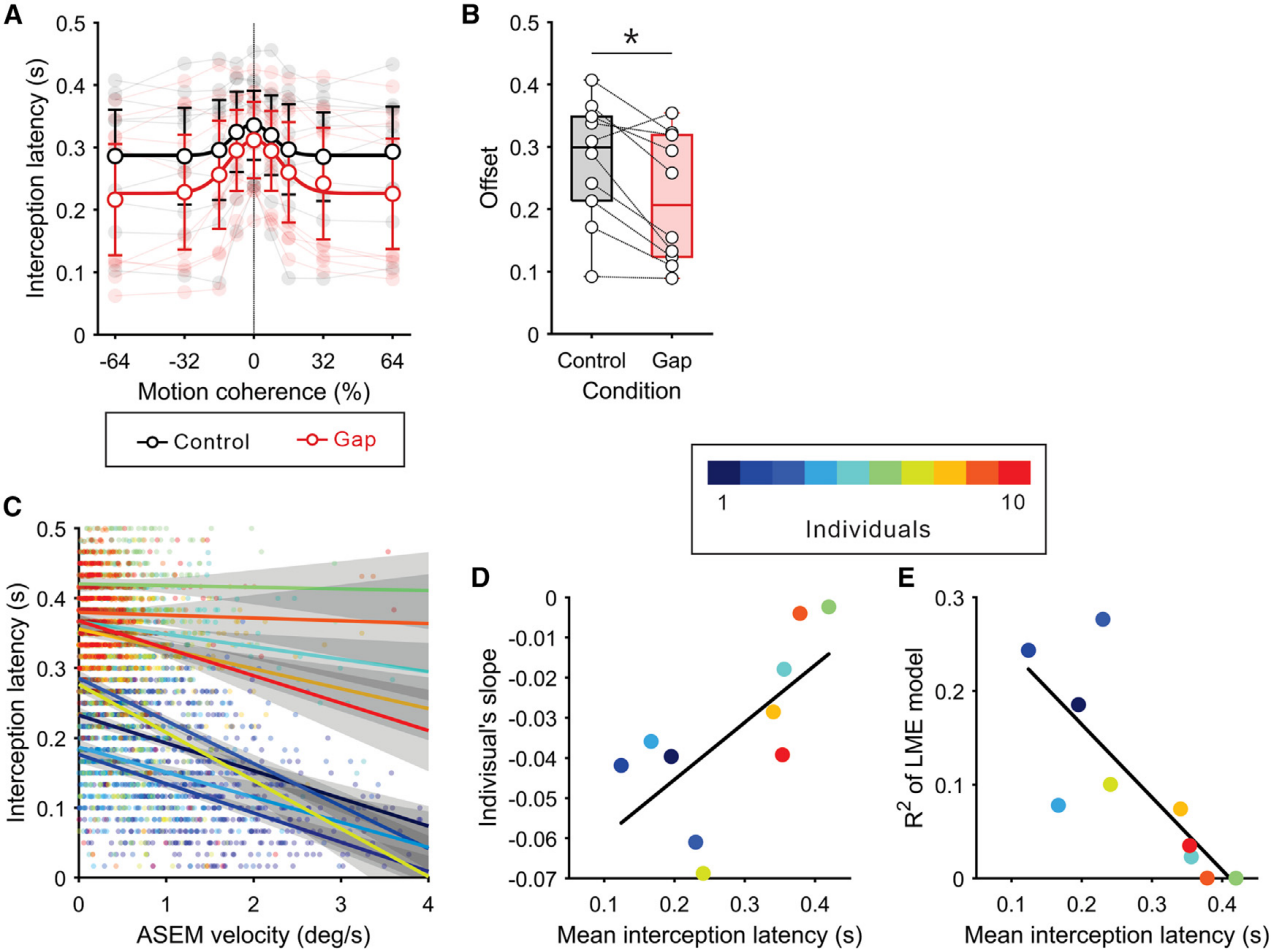
Interception latency and relationship to ASEM velocity (A) Interception latency as a function of motion coherence. Data are represented as mean and 95% confidence interval. Pale lines and dots in the background show individuals’ data. Solid curves indicate Gaussian functions fitted to the mean data for each condition. (B) Offset of Gaussian function shown in Figure 3A. Circles connected by dotted lines denote individuals’ data. An asterisk indicates a significant difference between conditions (paired *t* test: *t*_9_ = 3.40, *p* = 7.90 × 10^−3^, Cohen’s *d* = 0.65). (C) Fitted lines by linear mixed-effects model (3,509 trials from 10 participants) incorporating ASEM velocity and individual intercepts as random effects (formula: interception latency ∼ ASEM velocity + (1 + ASEM velocity | participant)). Each color of lines and pale dots corresponds to individuals. Shading shows 95% confidence intervals. (D) Individuals’ slopes of regression as a function of mean interception latency. Colors correspond to individuals (same as Figure 3C). The correlation coefficient *r* was 0.66 (*p* = 3.93 × 10^−2^). (E) Individuals’ explained variance (*r*^2^) of regression as a function of mean interception latency. Colors correspond to each participant (same as Figure 3C). The correlation coefficient *r* was −0.79 (*p* = 6.90 × 10^−3^).

Among the properties of interception movements, the latency of interception movements varied depending on the coherence level. Specifically, the interception latency increased when prediction certainty was low, as modeled by a Gaussian curve for motion coherence levels, with mean *r*^2^ values of 0.64 (SD: 0.20) for the control condition and 0.78 (SD: 0.13) for the gap condition (Figure 3A). Interestingly, the interception latency was shorter in the gap condition compared to the control condition (302.5 ± 96.7 ms for the control condition; 258.9 ± 108.8 ms for the gap condition, mean ± SD), as reflected in the offset of the Gaussian curves (*t*_9_ = 3.40, *p* = 7.90 × 10^−3^, Cohen’s *d* = 0.65; Figure 3B). The mean latency for the control condition was comparable to typical reaching reaction times reported in previous studies.^21^^,^^38^^,^^39^ Some participants showed shorter interception latencies compared to the typical reaching reaction time, likely due to their knowledge of the target motion onset timing. Thus, the interception latency here should not be interpreted as reflecting pure sensorimotor delays but rather as the onset of interception movements under uncertainty regarding the spatial location of the target presented at a predictable timing.^40^ Other properties of interception movements (Figure S2), including movement time, initial direction of interception, endpoint error, peak velocity in the x and y axes, and initial acceleration in the x and y axes, showed no effects related to the ASEM condition in two-way repeated-measures ANOVA (see the caption of Figure S2 for detailed statistical results). However, all properties except endpoint error showed significant main effects of coherence level in two-way repeated-measures ANOVA (see the caption of Figure S2 for detailed statistical results). These effects appeared to depend on the interception direction rather than coherence levels themselves. For example, a trend was observed where interception to the right (positive coherence) compared to the left (negative coherence) resulted in longer movement times and greater y-accelerations. This may be attributed to the positional relationship between the used hand (right) and the target. Indeed, none of the interception properties showing significant main effects of coherence level exhibited substantial differences across coherence levels within the same direction, as confirmed by *post hoc* tests using the Holm correction. These results suggest that facilitating ASEMs reduces interception latency while maintaining hand trajectory.

### Relationship between ASEM velocity and interception latency

The finding that ASEMs reduce interception latency is supported by evidence linking saccadic and reaching reaction times during eye-hand coordination tasks.^11^^,^^21^^,^^22^^,^^23^ Moreover, it is well established that eye movements can, in and of themselves, aid in the planning of hand movements.^10^^,^^18^^,^^19^^,^^41^ Based on these studies, we tested a quantitative relationship between ASEM velocity and interception latency using linear mixed-effects (LME) models with data from all single trials for both conditions. Prior to model fitting, ASEM velocity in each trial was re-signed so that the direction of the target motion was positive. We evaluated three models with interception latency as the dependent variable and ASEM velocity as a fixed effect: one with ASEM velocity and individual intercepts as random effects, another with only individual intercepts as random effects, and a third with a fixed intercept (no random effect). The model with ASEM velocity and individual intercepts as random effects was selected based on the Bayesian information criterion (BIC) (BIC values for the first, second, and third models were −8,674.5, −8,625.4, and −5,583.2, respectively). This model revealed a significant fixed effect of ASEM velocity (estimate ± SEM = −0.03 ± 0.01, *t*_3507_ = −4.32, *p* = 1.56 × 10^−5^) with *r*^2^ value of 0.71 (Figure 3C). These results suggest a substantial effect of ASEM velocity on interception latency, including individual differences. The individual differences in the relationship between ASEM velocity and interception latency may reflect characteristics of each participant’s interception. Indeed, as seen in Figure 3C, participants with shorter interception latencies (i.e., smaller y intercepts) appeared to exhibit steeper regression slopes. This assumption was corroborated by the correlation coefficient between the slopes and the mean interception latency for each participant (*r* = 0.66, *p* = 3.93 × 10^−2^; Figure 3D). Additionally, the variance explained in each participant was negatively correlated with the mean interception latency (*r* = −0.79, *p* = 6.90 × 10^−3^; Figure 3E), indicating that, in participants with shorter latencies, the onset timing of their interception more strongly depended on ASEM velocity.

Although we ensured that the temporal cue for target motion onset was equivalent between the control and gap conditions by presenting the square surrounding the fixation spot, we cannot completely rule out the possibility that the disappearance of the fixation spot had specific effects on the relationship between ASEM velocity and interception latency in the gap condition. To address this possibility, we conducted additional analyses on the data from the control condition. If the relationship between ASEM velocity and interception latency was solely a consequence of the fixation release, the relationship should not persist in the control condition where the fixation spot remained visible. Accordingly, we ranked trials within each participant according to ASEM velocity for each coherence level in the control condition and picked the upper and lower 20%. As expected, trials in the upper 20% of ASEM velocity exhibited shorter interception latencies compared to trials in the lower 20% (*t*_9_ = 2.48, *p* = 3.50 × 10^−2^, Cohen’s *d* = 0.24; Figure S3A). Furthermore, the results of the LME analysis could be reproduced even when restricted to data from the control condition (Figures S3B and S3C). These results confirm that the relationship between ASEM velocity and interception latency is not an artifact of the gap effect but rather evidence of the facilitative effect of ASEMs on initiating interception movements.

## Discussion

Contrary to existing evidence of the flexible regulatory system for ASEMs,^2^^,^^6^^,^^7^^,^^8^^,^^42^^,^^43^^,^^44^ their impact on goal-directed motor actions was previously unexplored. This study is the first to establish a direct link between ASEMs and manual interception, demonstrating the functional benefit of ASEMs in controlling rapid visually guided behaviors. Importantly, our result is not attributable to the predictive processes related to target motion, which are commonly involved in facilitating the initiation of eye and hand movements.^24^^,^^25^^,^^28^^,^^29^ Despite the partial involvement of the gap effect in our results,^45^ the observation that the relationship remains consistent under the control condition highlights a specific impact of ASEMs on interception latency. This effect seems distinct from the role of smooth pursuit in eye-hand coordination, which is typically associated with continuously sampling visual information and extrapolating object trajectories after the object’s disappearance.^13^^,^^14^^,^^15^ Instead, our results suggest a function of ASEMs similar to that of saccades, where eye reaction times correlate with hand reaction times in eye-hand coordination.^11^^,^^21^^,^^22^^,^^23^ Given that eye-hand coordination is consistently maintained across both saccades and smooth pursuit,^20^ it is not surprising that we observe similar effects of different types of eye movements on interception.

In this study, we used ASEM velocity as a representative measure to evaluate ASEM response, as it is a well-established index.^26^^,^^27^^,^^33^ However, as observed in the relationship between saccades and reaching movements,^11^^,^^21^^,^^22^^,^^23^ it may be more intuitive to assess the relationship between the reaction times of the eyes and hands (i.e., ASEM onset timing and interception latency in this study). The primary reason for using ASEM velocity was that detecting a reliable ASEM onset at the single-trial level was methodologically challenging due to the small amplitude of ASEMs (Figures 2A and 2B). It is important to note, however, that by definition, ASEM velocity in this study reflects both the timing of ASEM onset and the magnitude of its amplitude. Therefore, a large ASEM velocity was expected to correlate with an early ASEM onset. Furthermore, ASEMs generally begin earlier and become more pronounced as the target motion is repeated.^46^^,^^47^^,^^48^ This simultaneous progression can be explained by the buildup of reliability in extraretinal signals.^6^ Thus, if the extraretinal signals involved in ASEM control contribute to reducing interception latency, it should be possible to predict the latency based on the ASEM velocity computed in this study.

Our data suggest that the earlier onset of interception movements is primarily attributed to the extraretinal signals. The suggestion is supported by the observation that the relationship between ASEM velocity and interception latency depended on each participant’s mean interception latency. In this study, the mean interception latency across both conditions ranged from 120 to 420 ms. This range included latencies comparable to and much shorter than typical reaction times for reaching movements,^21^^,^^38^^,^^39^ indicating that the contribution of predictive versus reactive control to interception initiation varied among the participants. Importantly, participants with shorter interception latencies (i.e., those likely relying more on predictive control) exhibited a higher sensitivity of interception latency to ASEM velocity and a greater proportion of the variance in interception latency explained by ASEM velocity (Figures 3D and 3E). In contrast, in participants with interception latencies closer to typical reaction times (i.e., those likely relying less on predictive control), ASEM velocity was no longer a factor in explaining their interception latency. These results suggest that ASEMs shorten the onset of interception movements by facilitating predictive control rather than reactive control dependent on retinal signals. While the involvement of extraretinal signals in hand movements has been well-documented in studies on rapid interception movements with saccades^18^^,^^19^^,^^49^ and continuous hand tracking with smooth pursuit,^41^^,^^50^ their role in rapid manual interception during smooth pursuit remains unclear. Interestingly, a study on hand tracking has reported that restricting smooth pursuit delays the onset of hand movements.^51^ Taken together with the coupling between saccadic and hand reaction times,^21^^,^^22^ these findings suggest that motor signals to drive ASEMs may act as a trigger for initiating hand movements or facilitate a preparatory state for them.

In contrast to latency, facilitating ASEMs did not influence other properties of the interception movements. Additionally, these properties were largely unaffected by motion coherence except for positional constraints related to the used hand. These results indicate that the participants did not adjust their interception trajectory online based on prediction certainty about target motion but instead used a feedforward strategy aiming at the expected interceptive position. This approach seems reasonable for this task given that the target’s trajectory was predictable once it started moving, eliminating the need for online correction of their interception trajectory.^52^ However, previous studies on reaching strategies for multiple potential targets have reported a spatial averaging strategy, where observers aim at an intermediate direction between the potential targets to delay decision-making and accumulate more information before targeting uncertain locations.^40^ Since this strategy utilizes the probability of actions for each target as well as their spatial positions,^53^ the participants could have generated interception trajectories biased by the level of predictive certainty. However, the strategy is generally employed in slower movements where there is time for online correction of the interception trajectory, whereas rapid movements tend to aim directly at one of the potential targets.^54^ Given these studies, the participants in this study had access to the strategy but did not likely adopt it due to its low benefit in the current context.

Our results do not rule out the possibility that ASEM-induced changes in retinal signals could influence rapid interception movements in other contexts. In situations where retinal signals are crucial for online correction to the interception trajectory, such as when the target exhibits unpredictable motion, ASEMs may modulate the trajectory by altering the perception of the target motion. Our previous research has demonstrated that ASEM-induced decreases in retinal slip velocity during the initial phase of target motion lead to an underestimation of target velocity.^9^ However, there is also evidence suggesting that ASEMs do not bias the perception of target motion direction.^27^ The discrepancy between these findings may be partially attributed to differences in experimental paradigms used to induce ASEMs. Nevertheless, if motion perception parameters essential for achieving the desired interception movements (e.g., target velocity or direction) are altered by ASEMs, the resulting perceptual bias could modulate the trajectory accordingly.

Studies on the coordination of smooth pursuit and interception movements are limited, and the neural circuits responsible for their coupling are unidentified. Here we discuss candidate areas for interactions between ASEMs and initiation of interception movements based on studies on ASEM control and eye-hand coordination during saccades. The supplementary eye field (SEF) is a crucial area in cognitive control of ASEMs.^55^ Stimulation of the SEF facilitates ASEM responses when delivered just before target motion onset but does not induce eye movements when observers lack expectation of the target motion or when timing is irrelevant to the target motion onset.^56^^,^^57^ Since only a small population of SEF neurons is directly involved in generating motor responses, the SEF is believed to play a role in planning and preparation of ASEMs.^43^ The SEF has projections to the premotor cortex,^58^ which is involved in the preparation of limb movements. A study using a delayed reaching task has demonstrated that preparatory activity of neurons in the premotor cortex at the go cue correlated with reaching reaction times.^59^ Moreover, disruption of preparatory activity by microstimulation delays reaching reaction times.^60^ Given these studies, ASEM-related signals in SEF neurons may enhance the preparatory activity of neurons in the premotor cortex, thereby reducing interception latency. Indeed, disruption of preparatory activity in the premotor cortex affects only reaching reaction times but no other properties of reaching movements,^59^ consistent with our results. Another candidate is the superior colliculus (SC). Microstimulation of the deep layer of the SC, which receives projections from the SEF,^58^ induces target-directed reaching movements.^61^ Additionally, the posterior parietal cortex (PPC), including the parietal reach region and the lateral intraparietal area, has been extensively researched in the context of eye-hand coordination.^10^^,^^62^^,^^63^ However, while the PPC and SC are involved in the control of smooth pursuit directly or indirectly,^64^^,^^65^ their primary role is saccadic control. Additionally, several studies on eye-hand coordination suggest that the function of the SC is related to target selection for general actions,^66^ and the PPC encodes goals and associated coordinate transformations for saccades and reaching.^62^ The remaining challenges are to determine whether findings from studies on saccades are applicable to smooth pursuit and to identify the neural circuits involved in the control of ASEMs and interception movements.

### Limitations of the study

Our findings were derived from a relatively simple interception task and should be interpreted cautiously when applied to other contexts. Specifically, this study did not provide feedback on hand (cursor) trajectories and interception performance to participants, which can be crucial for visually guided interception strategies^52^^,^^67^ and learning or adaptation of motor control. Additionally, although the ASEM condition did not show a substantial effect on interception error in this study, facilitating ASEMs may influence interception performance in situations where latency is crucial for accuracy (e.g., high target velocities). Future studies should explore how ASEMs contribute to optimizing rapid interception performance in more naturalistic environments. Another limitation of this study is that the influence of sex and gender was not examined. To date, no study has reported their impact on ASEM properties. However, given that some studies have demonstrated sex differences in smooth pursuit performance,^68^ visual motion detection,^69^ and visuomotor tracking,^70^ participants’ sex could be a critical biological variable influencing ASEM responses and their impacts on visually guided behaviors.

## Resource availability

### Lead contact

Requests for further information and resources should be directed to and will be fulfilled by the lead contact, Takeshi Miyamoto (miyamoto@i.nagoya-u.ac.jp)

### Materials availability

This study did not generate new unique reagents or other materials.

### Data and code availability

• All data have been deposited at Zenodo at https://doi.org/10.5281/zenodo.14065281 and are publicly available as of the date of publication.
• All original code has been deposited at Zenodo at https://doi.org/10.5281/zenodo.14065281 and is publicly available as of the date of publication.
• Any additional information required to reanalyze the data reported in this paper is available from the lead contact upon request.

## Acknowledgments

This study was supported by Grants-in-Aid (KAKENHI) from the 10.13039/501100001691Japan Society for the Promotion of Science (JSPS) awarded to T.M. (grant no.: 22KJ1787 and 23K16671) and S.O. (grant no.: 23K24749) and by Open Access Acceleration Project.

## Author contributions

Conceptualization, T.M., K.N., and S.O.; methodology, T.M., K.N., and S.O.; software, T.M. and K.N.; formal analysis, T.M.; investigation, T.M., K.N., R.H., and Y.Y.; writing – original draft, T.M.; writing – review and editing, T.M. and S.O.; funding acquisition, T.M. and S.O.

## Declaration of interests

The authors declare no competing interests.

## STAR★Methods

### Key resources table

**Table:** 

REAGENT or RESOURCE	SOURCE	IDENTIFIER
**Deposited data**		

All data and analysis code	Zenodo	https://doi.org/10.5281/zenodo.14065281

**Software and algorithms**		

MATLAB (Version R2023a)	MathWorks, Natick, Massachusetts, USA	https://jp.mathworks.com/
Psychtoolbox 3	Braninard,^71^ Pelli,^72^ Kleiner et al.^73^	http://psychtoolbox.org/

### Experimental model and study participant details

Ten participants (7 men, 3 women) with normal or corrected-to-normal vision and no known visuomotor deficits participated in the study (age: 25.5 ± 2.6 years, mean ± SD). All participants were right-handed and diagnosed with neither stereoscopic problems nor strabismus. Sample sizes were not statistically predetermined but were comparable to those in previous studies on anticipatory smooth eye movements (ASEMs).^7^^,^^9^^,^^27^ They provided written informed consent in accordance with the Declaration of Helsinki and had the option to withdraw from the study at any time without penalty or explanation. The study protocol was approved by the Research Ethic Committee of the Faculty of Health and Sport Sciences, University of Tsukuba (approval number: 021–187).

### Method details

#### Materials

Participants were seated 57.0 cm from an LCD monitor (X27q QHD; HP Japan Inc., Tokyo, Japan; size: 27 inches, spatial resolution: 1440 × 2560 pixels, refresh rate: 60 Hz) with their heads stabilized by a chin rest and forehead restraint. The monitor was positioned vertically, with its height adjusted so that the participant’s eye level was aligned with a fixation spot during the task. The x and y axes in monitor coordinates were defined as the horizontal and vertical directions, respectively. Participants used a stylus on a digital tablet (Wacom Intuos Pro Large, Wacom Co., Ltd.; size: 311 × 216 mm) to maneuver an invisible cursor on the monitor. Cursor position data were sampled by MATLAB at 60 Hz.

Eye movements of the left eye were recorded using a video-based eye-tracking system. Eye position signals were detected via reflections of infrared light on the cornea, and the pupil’s image was captured by an infrared camera (GS3-U3-41C6NIR; FLIR Systems Inc., Oregon, USA).^74^ The system digitized eye position signals at 1 kHz with 16-bit precision using an A/D converter (Micro 1401; Cambridge Electronic Designs, Cambridge, UK). Prior to the task, eye position signals were calibrated by having participants fixate on target spots (diameter: 0.3 deg, luminance: 70.0 cd/m^2^) at known horizontal and vertical eccentricities under binocular viewing conditions. All stimuli during calibration and the main task were presented on a uniform black background (luminance: 0.1 cd/m^2^).

#### Experimental procedures

Each trial began with a gray fixation spot (0.2 × 0.2 deg) and a starting circle (diameter: 0.2 deg) positioned 15 deg below the fixation spot (Figure 1). Participants were instructed to gaze at the fixation spot throughout the trial while it was presented. They were required to place and maintain a magenta cursor (diameter: 0.1 deg) within the starting circle for 1.5 s before both the circle and cursor disappeared. After a random delay of 0.8–1.2 s, a random-dot motion (RDM) stimulus appeared within a 15 deg diameter aperture centered on the fixation spot. The purpose of the RDM was to encourage participants to make predictions about the target motion that would appear later in the trial, with various levels of prediction certainty. During the RDM stimulus, a subset of dots (2 × 2 pixels) moved either to the right or left, while other dots were replaced randomly. The stimulus was generated following protocols described in previous studies.^75^^,^^76^ Briefly, three interleaved sets of dots were drawn in successive frames. When a dot disappeared, it was redrawn three frames later either at a random location in the aperture or displaced in the direction of motion. The direction of the coherently moving dots indicated the direction of a later-appearing target, with the coherence level defining the level of prediction certainty. Coherence levels were selected from −0.64, −0.32, −0.16, −0.08, 0, 0.08, 0.16, 0.32, and 0.64 (negative and positive values indicating left and right directions, respectively). For RDM stimuli with coherence = 0, the target motion direction was assigned randomly. The dot density was 16.7 dots/deg^2^/s, and the coherent dots moved at a constant speed of 8.0 deg/s. The RDM stimulus and the fixation spot disappeared after 1.0 s, and following an additional 1.0 s blank period, the fixation spot reappeared in the same position. A target, a white ring with an outer diameter of 0.35 deg and an inner diameter of 0.27 deg,^7^ was presented slightly offset 1.5 s after the second fixation period and then moved in the same direction as the coherent motion of the RDM stimulus. Participants were instructed to intercept the target by manipulating the invisible cursor as quickly as possible while tracking the target with their eyes. They were asked to shoot the target without stopping to reach the target position. Two experimental conditions were set to either facilitate or suppress ASEMs: In the control condition, the fixation spot remained visible until the target motion onset, whereas in the gap condition, the last 300 ms of the period was replaced by a gap. This gap period, which immediately preceded the target motion onset, is known to facilitate ASEM responses.^30^^,^^31^^,^^32^ This gap potentially provided an additional cue regarding the timing of target motion onset in the gap condition (i.e., the disappearance of the fixation spot indicated that the target motion would begin 0.3 s later). To ensure that the available information regarding the target motion was the same across both conditions, we introduced a square (5.0 × 5.0 deg) surrounding the fixation spot, which was displayed in the second fixation period and then removed 0.3 s before target motion onset in all trials, mirroring the timing of the fixation spot disappearance in the gap condition. Each trial ended when the cursor passed the y axis position of the target or 1.2 s after the target motion onset, followed by a 2.0 s inter-trial interval. No feedback was provided to the participants. Trials in which the cursor left the starting circle before the target motion onset were discarded, and a warning message was shown.

Each participant completed a total of 360 trials (2 conditions × 9 coherence levels × 20 trials) interleaved and divided into five blocks. For familiarization, participants practiced the task for one block with the cursor visible. All visual stimuli and experimental routine were programmed using Psychophysics Toolbox extensions in MATLAB.^71^^,^^72^^,^^73^

### Quantification and statistical analysis

Trials were excluded if participants exhibited blinks during target motion, made saccades within a 50 ms window before and after target motion onset (i.e., ASEM epoch), initiated interception movements before target motion onset, or had interception latencies longer than 500 ms. Since the duration of the second fixation period was constant throughout the experiment (i.e., always 1.5 s after the fixation spot appeared), participants were aware of the precise timing of the target motion onset. As such, the interception latency in this study should be interpreted not as a pure sensorimotor delay, but rather as the onset of interception movements made under uncertainty about target motion, with a predictable timing. In line with this interpretation, trials with interception latencies shorter than typical reaching reaction times reported in previous studies^21^^,^^38^^,^^39^ were retained. A total of 3509 out of 3600 trials were included in the subsequent analyses (average retention rate of 97.5%).

Eye position data were filtered with a second-order Butterworth low-pass filter with a 15 Hz passband. Eye velocity and acceleration were calculated using digital differentiation of the position data with the central difference algorithm and then filtered with a second-order Butterworth low-pass filter with a 30 Hz passband. Saccades were identified based on criteria of acceleration exceeding 1000 deg/s^2^ and velocity exceeding 30 deg/s, and linear interpolation was used to fill gaps left by removed saccades. ASEM velocity was defined as the mean eye velocity within 50 ms before and after target motion onset (Figures 2A and 2B).^26^^,^^27^^,^^33^ To quantify and compare ASEM velocity as a function of prediction certainty, we computed the mean ASEM velocity for each condition × coherence combination and fitted a cumulative Gaussian function to the data for each condition:

(Equation 1) $$\begin{array}{c} ASEMvel=\frac{a}{2}\left(1+\mathrm{erf}\left(\frac{x-\mu }{\sigma \sqrt{2}}\right)\right)+b \end{array}$$

where x is the motion coherence, μ and σ are the Gaussian parameters, a controls the height of the curve, and b is the baseline (lower asymptote) (Figure 2C). The height parameter a was used to compare the amplitude of ASEM responses between the conditions (Figure 2D).

To investigate which of the decision outcome (i.e., left or right) or the confidence level in the perceptual decision regarding the RDM stimulus determined ASEM velocity, we examined the distribution of ASEM velocity across all participants. If ASEM velocity were determined by the outcome of their decision, we would expect a bimodal distribution. Conversely, if ASEM velocity were determined by the decision confidence, the distribution would remain unimodal but shift according to the level of prediction certainty. We z-normalized ASEM velocity for each participant across all trials and then pooled the data across all participants. The cumulative frequency distribution was calculated for each combination of the ASEM condition and motion coherence (Figure 1C).

Cursor position data were filtered with a second-order Butterworth low-pass filter with a 20 Hz passband, and hand velocity and acceleration were calculated similarly to eye data. Interception onset was defined as the time when the cursor velocity exceeded 1.0 deg/s. Interception latency was the time interval between the target and interception onset. Initial hand accelerations in the x- and y axes were defined as the mean acceleration during the first 100 ms after target motion onset. Initial direction of interception was calculated as the angle between the midline and the vector linking the cursor position at the start to the cursor position at peak acceleration.^77^ Endpoint error was defined as the absolute distance in the x axis between the target and the cursor when the cursor reached the target’s y-position.

To quantify and compare interception latency as a function of prediction certainty, we fitted a Gaussian function to the mean interception latency according to motion coherence for each condition:

(Equation 2) $$\begin{array}{c} Latency=c\cdot \exp\left(\frac{-{\left(x-\mu \right)}^{2}}{2\sigma^{2}}\right)+d \end{array}$$

where c is the Gaussian peak amplitude and d is the constant offset (Figure 3A). The offset parameter d was used to compare interception latency between the conditions (Figure 3B).

If interception movements were modulated by ASEMs, some of the interception properties should vary depending on the experimental facilitation or inhibition of ASEMs (i.e., the gap and control conditions, respectively). To confirm that the ASEM response was conditioned and to test the impact of the ASEM condition on interception movements, we performed statistical comparisons using paired *t* tests or two-way repeated-measures analysis of variance (rmANOVA). Specifically, paired *t* tests were used to examine the effect of the ASEM condition on the amplitude parameters of ASEM velocity and interception latency, while rmANOVA was applied to other interception properties to assess the effects of the ASEM condition and motion coherence level. Significant results from the rmANOVA were followed up with post-hoc multiple comparisons using the Holm correction. All statistical tests were conducted at a significance level of 0.05. Significance of *t* tests is denoted with a single asterisk in Figures 2E, 3B, and S3A.

Among the interception properties, the interception latency showed a significant effect of the ASEM condition. Therefore, we further tested a quantitative relationship between ASEM velocity and interception latency using linear mixed-effects (LME) models. ASEM velocity for all trials was re-signed so that the direction of target motion was positive before being used in the LME models. Three models with interception latency as the dependent variable and ASEM velocity as a fixed effect were computed and compared based on the Bayesian Information Criterion (BIC): one with ASEM velocity and individual intercepts as random effects [formula: interception latency ∼ ASEM velocity + (1 | participant) + (1 + ASEM velocity | participant)], another with only individual intercepts as random effects [formula: interception latency ∼ ASEM velocity + (1 | participant)], and a third with a fixed intercept [formula: interception latency ∼1 + ASEM velocity]. The LME procedure was also applied to the data within each condition.

Since the model with a random intercept was adopted in the LME analysis, we further computed Pearson’s correlation coefficients between the regression slope, the explained variance, and the mean interception latency for each participant. These analyses aimed to determine whether individual differences in the relationship between ASEM velocity and interception latency reflected characteristics of each participant’s interception.

All analyses, including statistical tests, were conducted using MATLAB. Detailed statistical results are provided in the results section and/or figure legends.
