# Supplementary material for: Reduced latency in manual interception with anticipatory smooth eye movements
Source: iScience. 2025 Jan 20;28(2):111849. doi: 10.1016/j.isci.2025.111849 (PMC11834127; doi:10.1016/j.isci.2025.111849)
Supplement: Document S1. Figures S1–S3 [file mmc1.pdf]

iScience, Volume *28*

Supplemental information

Reduced latency in manual interception

with anticipatory smooth eye movements

Takeshi Miyamoto, Kosuke Numasawa, Riku Hirano, Yusei Yoshimura, and Seiji Ono

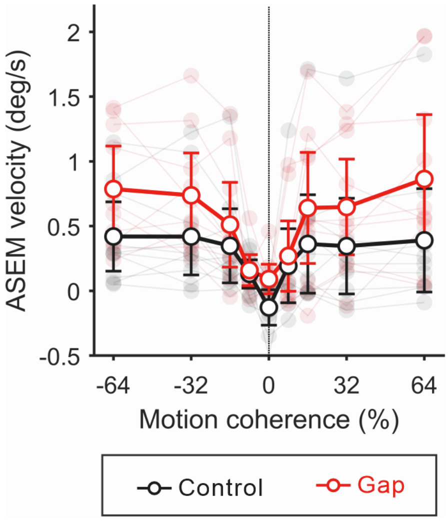
ASEM velocity (deg/s)
2
1.5
1
0.5
0
-0.5
-64
-32
0
32
64
Motion coherence (%)
Control
Gap

Figure S1

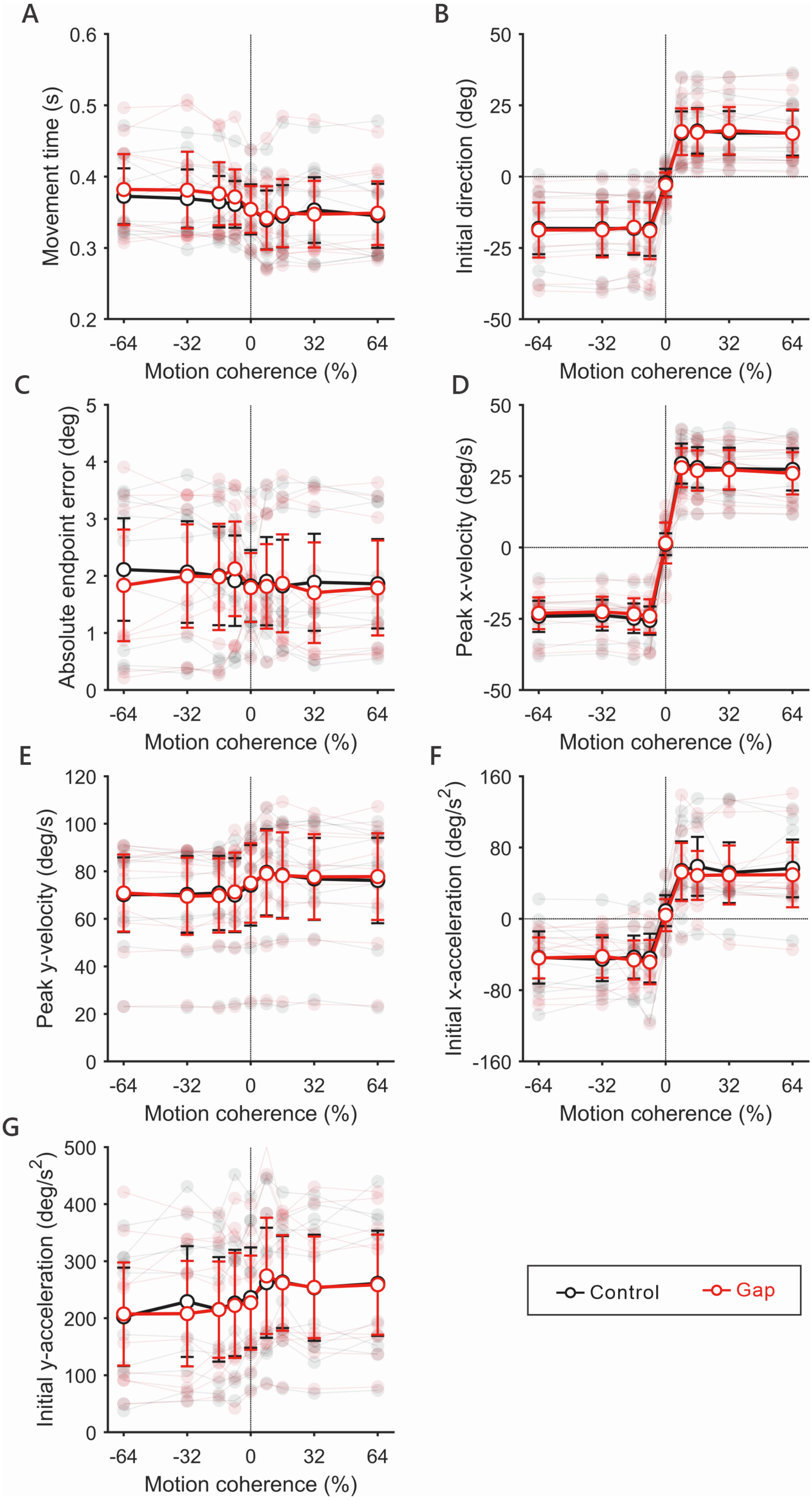

A
B
C
D
E
F
G
Movement time (s)
Initial direction (deg)
Absolute endpoint error (deg)
Peak x-velocity (deg/s)
Peak y-velocity (deg/s)
Initial x-acceleration (deg/s^2)
Initial y-acceleration (deg/s^2)
Motion coherence (%)
-64
-32
0
32
64
Control
Gap

Figure S2

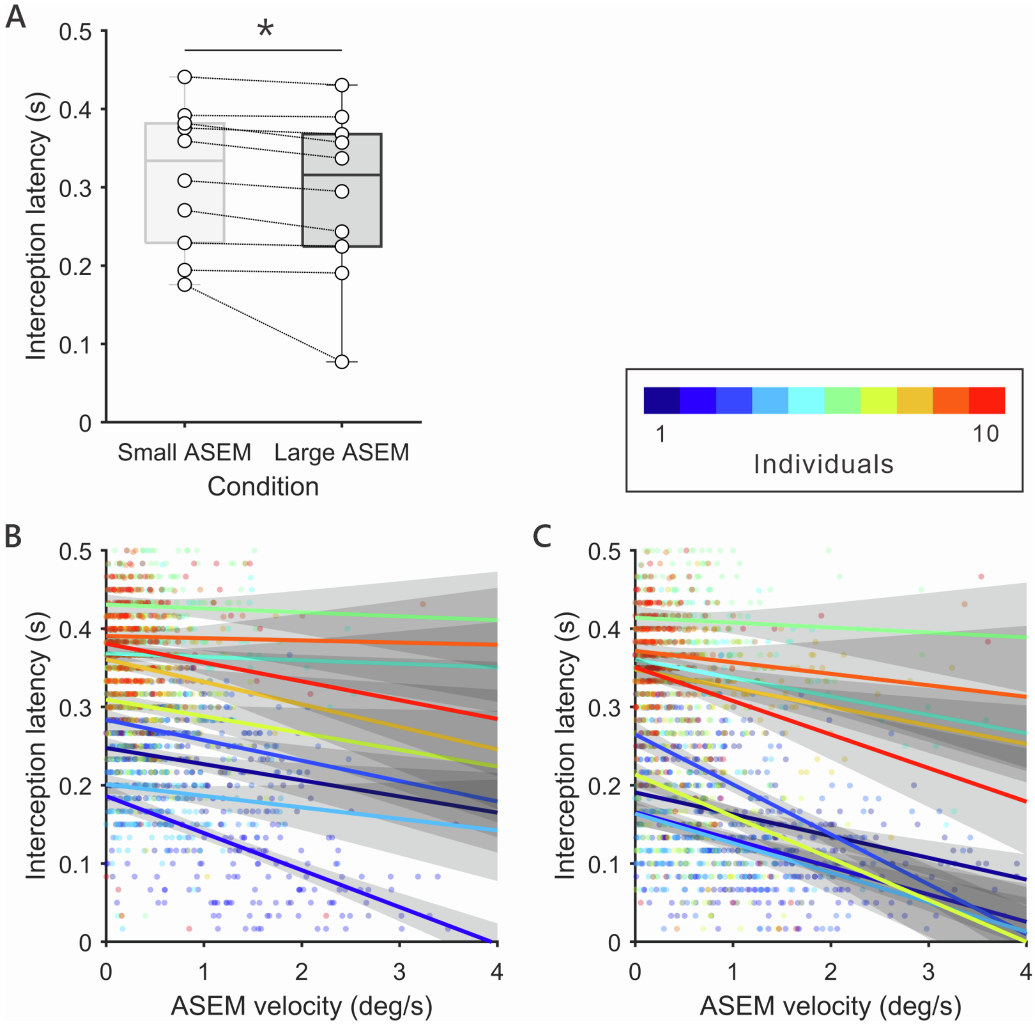

A
*
Interception latency (s)
0.5
0.4
0.3
0.2
0.1
0
Small ASEM
Large ASEM
Condition
1
10
Individuals
B
C
Interception latency (s)
ASEM velocity (deg/s)
0
1
2
3
4

Figure S3

SUPPLEMENTARY FIGURE LEGENDS

Figure S1. ASEM velocity in target motion direction. ASEM velocity in target motion direction as a function of motion coherence. Circles denote mean values across participants, and error bars denote 95% confidence intervals.

Figure S2. Other properties of interception movement behaviors. (A) Movement time as a function of motion coherence. Data are represented as mean and 95% confidence interval. Pale lines and dots in the background show individuals' data. Solid curves indicate cumulative Gaussian functions fitted to the mean data for each condition. Two-way rmANOVA showed a main effect of coherence level ($F_{8,72} = 5.19$, $p = 3.93 \times 10^{-5}$, $\eta_p^2 = 0.37$) but neither a main effect of ASEM condition ($F_{1,9} = 4.00$, $p = 0.08$, $\eta_p^2 = 0.31$) nor interaction ($F_{8,72} = 1.33$, $p = 0.24$, $\eta_p^2 = 0.13$). (B) Initial direction of interception as a function of motion coherence, plotted in the same format as Figure S2A. Two-way rmANOVA showed a main effect of coherence level ($F_{8,72} = 21.91$, $p = 1.72 \times 10^{-16}$, $\eta_p^2 = 0.71$) but neither a main effect of ASEM condition ($F_{1,9} = 0.31$, $p = 0.59$, $\eta_p^2 = 0.03$) nor interaction ($F_{8,72} = 0.49$, $p = 0.86$, $\eta_p^2 = 0.05$). (C) Absolute endpoint error in the x-axis as a function of motion coherence, plotted in the same format as Figure S2A. Two-way rmANOVA showed none of main effects of coherence level ($F_{8,72} = 0.20$, $p = 0.99$, $\eta_p^2 = 0.02$) nor ASEM condition ($F_{1,9} = 0.30$, $p = 0.59$, $\eta_p^2 = 0.03$), and interaction ($F_{8,72} = 1.89$, $p = 0.07$, $\eta_p^2 = 0.17$). (D) Peak hand velocity in the x-axis as a function of motion coherence, plotted in the same format as Figure S2A. Two-way rmANOVA showed a main effect of coherence level ($F_{8,72} = 100.44$, $p = 6.23 \times 10^{-36}$, $\eta_p^2 = 0.92$) but neither a main effect of ASEM condition ($F_{1,9} = 0.39$, $p = 0.55$, $\eta_p^2 = 0.04$) nor interaction ($F_{8,72} = 1.26$, $p = 0.28$, $\eta_p^2 = 0.12$). (E) Peak hand velocity in the y-axis as a function of motion coherence, plotted in the same format as Figure S2A. Two-way rmANOVA showed a main effect of coherence level ($F_{8,72} = 10.43$, $p = 1.40 \times 10^{-9}$, $\eta_p^2 = 0.54$) but neither a main effect of ASEM condition ($F_{1,9} = 1.40$, $p = 0.27$, $\eta_p^2 = 0.13$) nor interaction ($F_{8,72} = 0.81$, $p = 0.60$, $\eta_p^2 = 0.08$). (F) Initial hand acceleration in the x-axis as a function of motion coherence, plotted in the same format as Figure S2A. Two-way rmANOVA showed a main effect of coherence level ($F_{8,72} = 23.80$, $p = 2.20 \times 10^{-17}$, $\eta_p^2 = 0.73$) but neither a main effect of ASEM condition ($F_{1,9} = 4.60$, $p = 0.06$, $\eta_p^2 = 0.34$) nor interaction ($F_{8,72} = 0.49$, $p = 0.86$, $\eta_p^2 = 0.06$). (G) Initial hand acceleration in the y-axis as a function of motion coherence, plotted in the same format as Figure S2A. Two-way rmANOVA showed a main effect of coherence level ($F_{8,72} = 5.80$, $p = 1.05 \times 10^{-5}$, $\eta_p^2 = 0.39$) but neither a main effect of ASEM condition ($F_{1,9} = 0.15$, $p = 0.71$, $\eta_p^2 = 0.02$) nor interaction ($F_{8,72} = 1.19$, $p = 0.32$, $\eta_p^2 = 0.12$). None of the interception properties showing significant main effects of coherence level (movement time, peak velocity in the x- and y-axes, and initial acceleration in the x- and y-axes) exhibited substantial differences across coherence levels within the same direction, as confirmed by post-hoc tests using the Holm correction.

Figure S3. LME models within each condition. (A) Comparison of interception latency between trials with small ASEM velocity (lower 20%) and trials with large ASEM velocity (upper 20%) within the control condition. Circles connected by dotted lines represent individuals' data. An asterisk indicates a significant difference between conditions (paired *t* test: $t_9 = 2.48$, $p = 3.50 \times 10^{-2}$, Cohen's $d = 0.24$). (B) Fitted lines for data in the control condition from the LME model (1742 trials from 10 participants) with ASEM velocity and individual intercepts as random effects ($r^2 = 0.71$). Each color of lines and pale dots correspond to individuals. The shading shows 95% confidence intervals. (C) Fitted lines for data in the gap condition from the LME model (1767 trials from 10 participants) with ASEM velocity and individual intercepts as random effects ($r^2 = 0.70$), plotted in the same format as Figure S3B.
